# A Landscape of Lignocellulosic Biopolymer Transformations into Valuable Molecules by Heterogeneous Catalysis in C’Durable Team at IRCELYON

**DOI:** 10.3390/molecules26226796

**Published:** 2021-11-10

**Authors:** Laurent Djakovitch, Nadine Essayem, Marion Eternot, Franck Rataboul

**Affiliations:** Université Claude Bernard Lyon 1, CNRS, IRCELYON, 2 Avenue Albert Einstein, CEDEX, 69626 Villeurbanne, France; laurent.djakovitch@ircelyon.univ-lyon1.fr (L.D.); nadine.essayem@ircelyon.univ-lyon1.fr (N.E.); marion.eternot@ircelyon.univ-lyon1.fr (M.E.)

**Keywords:** wood, cellulose, lignin, biomolecules, acid catalysis, metal supported catalysis

## Abstract

This review article highlights part of the research activity of the C’Durable team at IRCELYON in the field of sustainable chemistry. This review presents a landscape of the work performed on the valorization of lignocellulosic biopolymers. These studies intend to transform cellulose, hemicellulose and lignin into valuable molecules. The methodology usually consists in evaluating the behavior of the biopolymers in the absence of catalyst under various conditions (solvent, temperature), and then to assess the influence of a catalyst, most often a heterogeneous catalyst, on the reactivity. The most significant results obtained on the upgrading of cellulose and lignin, which have been mainly investigated in the team, will be presented with an opening on studies involving raw lignocellulose.

## 1. Introduction

The use of biosourced reactants for the production on molecules of interest is of definitive importance for the development of a green chemistry [1,2]. The C’Durable team at IRCELYON has been involved for many years in the valorization of various bioresources. We particularly use heterogeneous catalysis, which is a primordial tool to implement sustainable procedures [3,4,5,6]. Initial works in the team focused on upgrading first generation biomass like vegetable oils [7,8,9,10], starch [11,12,13,14], glycerol [15,16,17,18,19,20,21], simple saccharides [22,23,24,25,26,27,28,29,30,31], and their furan derivatives [32,33,34]. Studies involving second generation biomass, especially lignocellulose (wood and grassy plants), have started more recently and are now predominant. All the past and current research in this area has given to our team a certain notoriety within the French catalysis and green chemistry communities [35]. We would like to present here an overview of the research performed in the C’Durable team on the transformation of lignocellulosic biopolymers. We will focus on the molecular aspects of the catalytic transformations through the main outputs coming from recent projects. Most of them involved the valorization of single biopolymers constituting lignocellulose which are cellulose and lignin. The use of raw lignocellulose has started more recently; general strategies and perspectives for its catalytic transformation will also be presented.

## 2. Transformation of Cellulose

**Scheme 1:**
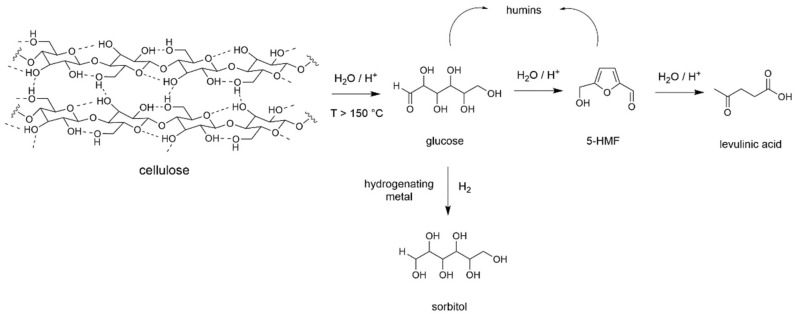
Cellulose depolymerization in the presence of a Brønsted acid.

Cellulose is the main component of lignocellulosic biomass [36]. It is a biopolymer composed of glucosyl units and the most straightforward molecular transformation of cellulose implies a depolymerization into glucose (Scheme 1). However, due to its structural arrangement made of a dense H-bonds network, cellulose is almost chemically inert and insoluble under usual conditions of solvent and temperature. If one excludes biotechnological methods, such transformation into light products requires demanding conditions, like solvothermal treatments in the presence of a catalyst [37]. Catalysts for depolymerization are usually Brønsted acids, either homogeneous (HCl, H_2_SO_4_, H_3_PO_4_) or heterogeneous. Several types of heterogeneous catalysts have been used like zeolites, heteropoly acids [38], but best results were obtained with sulfonated materials. Here, research groups of Zhang [39], Hara [40] and Onda [41] pioneered studies with sulfonated carbons while Jacobs, Sels et al. [42] introduced sulfonated silica and carbon nanocomposites giving glucose with yields over 50 mol%. Water is the adequate reaction medium due to compatibility with formed products and its green solvent character [43,44]. However, due to the reaction conditions to get significant conversion (temperatures over 150 °C on day scale), glucose is rarely formed as sole product and always reacts to give 5-hydroxymethylfurfural (5-HMF) then levulinic acid together with uncontrolled formation of soluble/insoluble oligomers known as humins (Scheme 1) [45]. To try to overcome these issues, non-conventional reaction media like ionic liquids [46], deep eutectic solvents [47], or activation methods like ball-milling, non-thermal plasma and high frequency ultrasound have been successfully introduced, even in the absence of acid catalyst, as demonstrated by Jérôme et al. [48].

When a hydrogenating metal like Pd, Pd, Ru or Ni is present within the catalytic system, and the depolymerization performed under H_2_, glucose is rapidly reduced to sorbitol (Scheme 1), another molecule of great interest. Here, Fukuoka et al. reported the first synthesis of sorbitol with 30 mol% yield over Pt/γ-Al_2_O_3_ catalyst [49]. Works from Ruppert and Palkovits groups did follow, leading to a variety of catalytic systems combining acid and metal functions increasing yields up to 70 mol% [50,51]. Besides, Zhang et al. showed that when the reaction was performed at a higher temperature and in the presence of supported Ni and tungsten carbides, C-C bond cleavages occurred leading to ethylene and propylene glycol with combined yield up to 65 wt% [52].

### 2.1. Transformation of Cellulose in Water

**Scheme 2:**
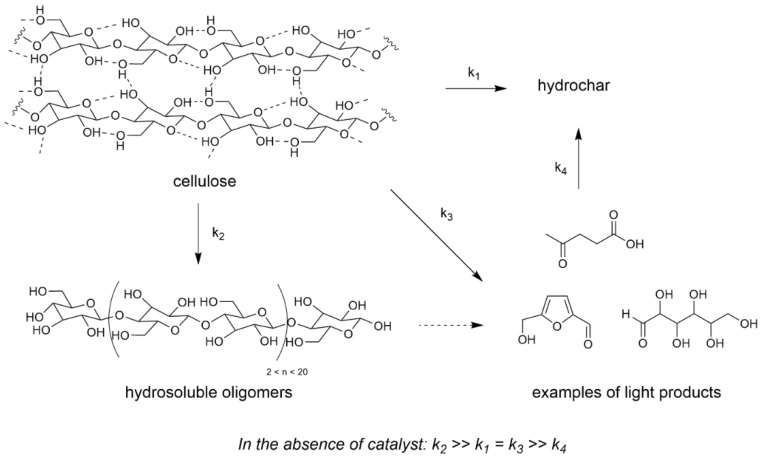
Reaction pathway for non-catalyzed cellulose liquefaction (water 60 mL, Avicel cellulose 0.2–4 g, 175–200 °C, 0–120 h). Adapted from [56].

When we initiated our research on cellulose transformation with heterogeneous catalysts, we were first interested in evaluating the non-catalyzed reactivity of cellulose in water, that was rarely investigated at that time. We observed for the first time that a reaction temperature of 190 °C was sufficient to liquefy (i.e., dissolve and/or convert) cellulose up to 60 wt% after 120 h, even in the absence of a catalyst [53]. This was possible thanks to the water dissociation at this temperature providing protons in the reaction medium [54]. Nevertheless the latter presented less than 15 wt% of deep depolymerization products (glucose, 5-HMF…). Therefore this non-catalyzed reaction formed preferentially what we name hydrosoluble oligomers and polymers (either as primary or as recondensation products) and hydrochar (Scheme 2) [55].

**Figure 1:**
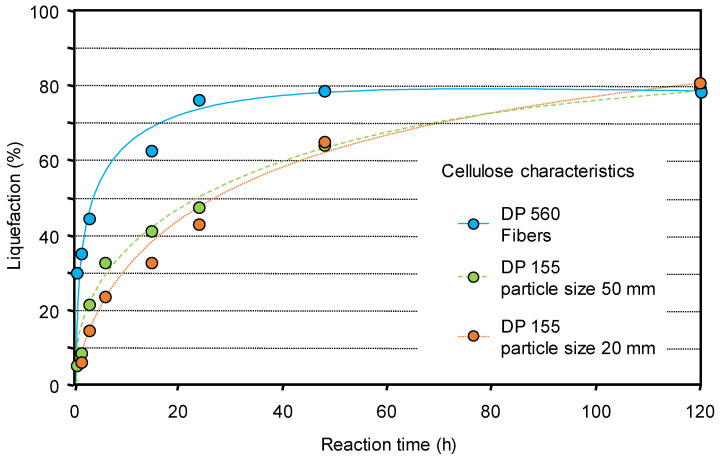
Non-catalyzed liquefaction of celluloses of different characteristics (water 60 mL, Avicel and Sigmacell celluloses 2 g, 190 °C). Liquefaction was determined by the difference between the initial and residual mass of cellulose. Adapted from [56].

Moreover it appeared that the intrinsic characteristics of the cellulose are a parameter influencing its reactivity and the morphology plays a role more important than polymerization degree or particle size. Celluloses with a fiber structure, despite a much higher polymerization degree (DP), react faster than those in particle forms (Figure 1) [56].

**Scheme 3:**
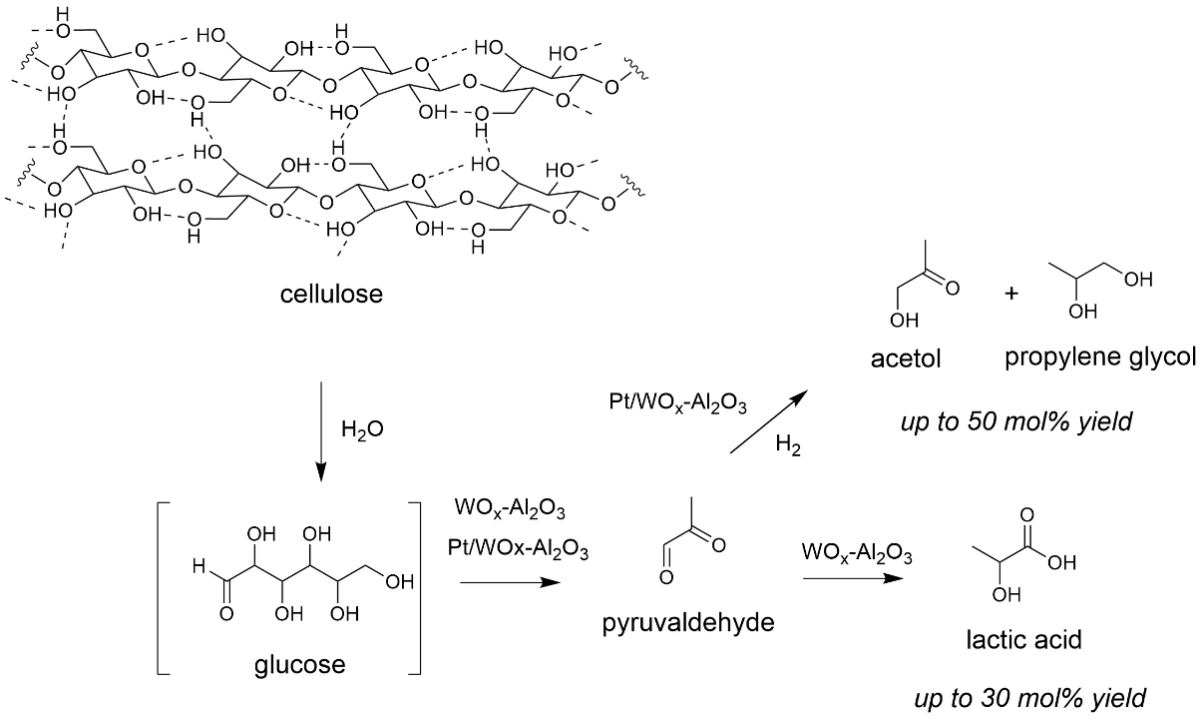
Reaction pathways of cellulose transformation in the presence of solid Lewis acids (water 65 mL, Avicel cellulose 1.6 g, catalyst 0.68 g, 190 °C, 5 MPa 24 h). Yield = 100 × (n_product_/n_glucosyl units_) × (3/6). Adapted from [60,64].

Then we assessed for the first time the behaviour of solid Lewis acids as catalysts for the depolymerization of cellulose [57,58]. We studied the reaction in the presence of tungstated zirconia WO_x_-ZrO_2_ and alumina WO_x_-Al_2_O_3_. Yields are determined based on the amount of moles of initial glucosyl units C_6_H_10_O_5_, (n_glucosyl units_ = m_dry cellulose_/162), corrected by the number of carbons in the product. We observed a different reactivity than that occurring with Brønsted acids (see Scheme 1). While the latter form preferentially levulinic acid, Lewis acids formed lactic acid as main product up to 30 mol% yield [59,60], a possible monomer for biosourced plastics. This corresponds to the first report on the formation of lactic acid from cellulose, even more with a water-tolerant heterogeneous catalyst [61]. Indeed, studies reported at the same time by Taarning et al. described the formation of lactic acid with zeolites from monomeric sugars only [62]. The reaction mechanism supposedly involves the presence of vacant Lewis sites at the surface of the catalyst able to promote dihydroxylation and carbon-carbon bond cleavages of the glucose chains in addition to hydride transfers giving pyruvaldehyde as intermediate [60]. Note that when lignocellulose was used as reactant in place of cellulose in same conditions, very close results were obtained, showing that the presence of other wood components may have no impact on the transformation of cellulose into lactic acid (see Section 4 for more details) [63]. When WO_x_-ZrO_2_ or WO_x_-Al_2_O_3_ served as support for Pt, and the transformation performed under H_2_, we observed the reduction of the intermediate to acetol and propylene glycol through successive hydrogenation steps (Scheme 3) [64,65].

**Scheme 4:**
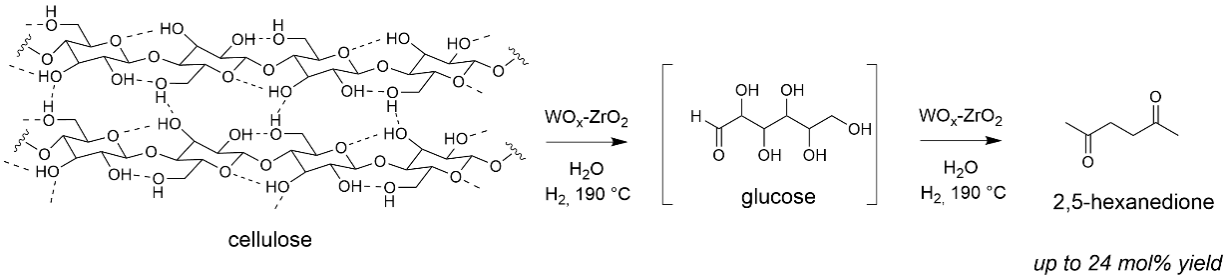
Formation of 2,5-hexanedione from cellulose in the presence of solid Lewis acid (water 65 mL, Avicel cellulose 1.6 g, catalyst 0.68 g, 190 °C, 5 MPa 24 h). Yield = 100 × (n_product_/n_glucosyl units_) × (6/6). Adapted from [66].

Interestingly, in the absence of Pt but still under H_2_ atmosphere, another different route occurred forming 2,5-hexanedione as main product (Scheme 4) with WO_x_-ZrO_2_ [66]. This was possible from hydroxyl groups abstraction promoted by this catalyst under the hydrothermal conditions as proven by quantitative Infra-Red spectroscopy analysis and kinetic investigations [67]. This highlights the different mode of action of WO_x_-ZrO_2_ vs. WO_x_-Al_2_O_3_ in the depolymerization of cellulose. That was the first report on direct 2,5-hexanedione formation from cellulose, a molecule of high interest for green fuels aviation synthesis by aldol condensation [68].

### 2.2. Transformation of Cellulose in Liquid and Supercritical Organic Solvents

The reactivity of cellulose in alcohols was studied with the aim of forming levulinic esters as high-interest biosourced molecules for energy or chemical applications [69]. The alcohol serves as reaction medium and esterifying agent. Levulinic esters are usually obtained by esterification of levulinic acid in the presence of a variety of catalytic systems; however, the possibility of forming these compounds from cellulose has been previously demonstrated by Garves, however, without real further investigation since then [70].

**Figure 2:**
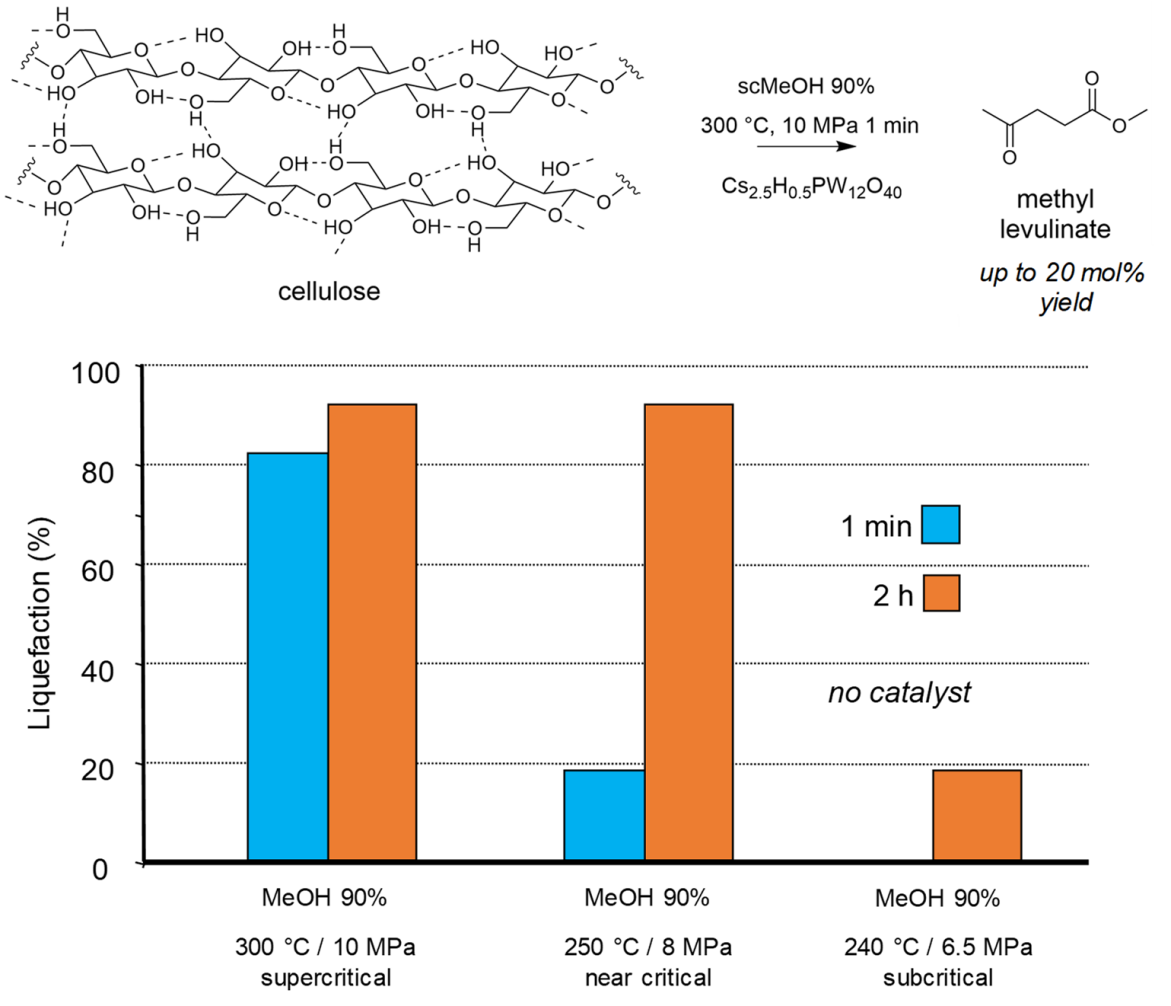
Cellulose transformation into methyl levulinate in supercritical MeOH (methanol 90 wt% 25 g, cellulose 0.35 g, catalyst if present 0.15 g). Yield = 100 × (n_product_/n_glucosyl units_) × (5/6). Adapted from [71].

We showed that reacting cellulose in methanol under supercritical conditions led to the deep liquefaction within few minutes, even in the absence of catalyst. The supercritical conditions were necessary to get this very fast reactivity (Figure 2). The presence of the solid Brønsted acid Cs_2.5_H_0.5_PW_12_O_40_, formed as expected methyl levulinate in 20 mol% yield [71]. For such reactions the presence of 10 wt% of water in methanol enhanced the overall reactivity.

**Figure 3:**
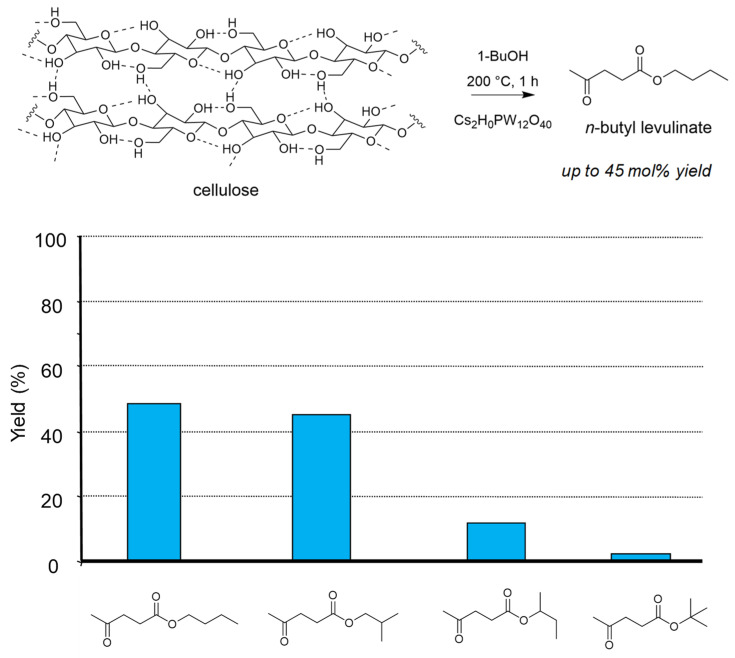
Cellulose transformation into butyl levulinates in butanol isomers (butanol 20 g, cellulose 0.5 g, H_2_SO_4_ 0.125 g, 200 °C, 0.5 h). Yield = 100 × (n_product_/n_glucosyl units_) × (5/6). Adapted from [72].

The reaction was extended to butanol to form butyl levulinates, and in this case we did not observe the influence of these supercritical conditions on the global reactivity. However, we demonstrated the importance of the butanol isomer on the formation of the corresponding butyl levulinate. Here the primary butanols gave the best yields up to 50 mol%, both in the presence of H_2_SO_4_ or Cs_2_HPW_12_O_40_ (Figure 3) [72]. Note that tert-butyl levulinate was formed as traces due to fast dehydration of tert-butanol preventing its role of esterifying agent.

**Scheme 5:**
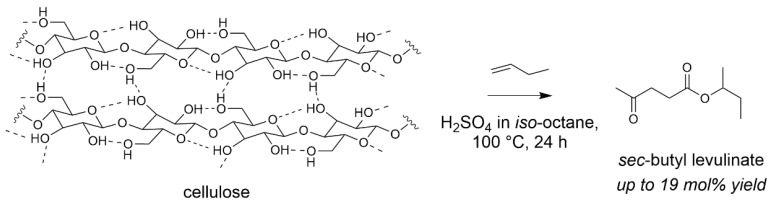
Cellulose transformation into butyl levulinates with olefins (iso-octane 50 mL, Avicel cellulose 0.5 g, 1-butene 3.4 g, H_2_SO_4_ 0.1 g, 100 °C, 24 h). Yield = 100 × (n_product_/n_glucosyl unit_) × (5/6). Adapted from [74].

With the aim of avoiding water formation during the esterification step with a more efficient atom utilisation, we assessed butenes as esterifying agent. Previous study has been reported by Dumesic et al. forming butyl levulinates in two-step procedures through reactive extraction in the presence of sulfuric acid [73]. In our case, the challenge was to find conditions able to accommodate all reagents for a one-step transformation. With the use of H_2_SO_4_ in an organic solvent, iso-octane, we were able to form a levulinic ester from the reaction of cellulose with an olefin (Scheme 5). Sec-butyl levulinate was formed in a 19 mol% yield, higher than using the corresponding alcohol. Attempts to change iso-octane to a biosourced solvent, γ-butyrolactone, led to the formation of sec-butyl levulinate, with a 5 mol% yield. This confirms the difficulty to find suitable conditions for a highly sustainable procedure [74].

**Scheme 6:**
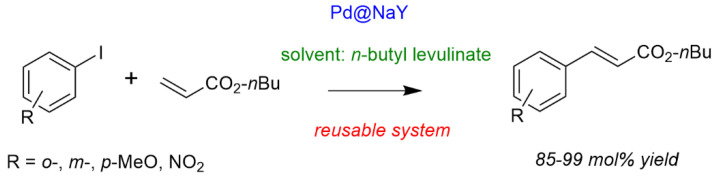
Heck coupling in n-butyl levulinate as solvent (solvent 6 mL, aryl halide 6 mmol, n-butyl acrylate 7 mmol, NEt_3_ 6.6 mmol, Pd 6 μmol, 150 °C, 6 h). Yield_product_ = 100 × (n_product_/n_Ar-I_). Adapted from [76].

Finally, as a chemical application of butyl levulinates, with showed for the first time their use as biosourced solvents for synthetic chemistry like C-C couplings. Vaccaro et al. previously demonstrated the ability of γ-valerolactone (GVL) as biosourced solvent for this chemistry with therefore the potential to replace (toxic) organic solvents generally used for such transformations [75]. However, since alkyl levulinates are the precursors of GVL, it would be more interesting to use them directly as solvents. Here, n-butyl levulinate showed to be as suitable as GVL or organic solvents for Heck C-C coupling, even in the presence of a heterogeneous catalyst. The system solvent/catalyst has shown to be completely recoverable and reusable compiling many requirements for a green synthetic route (Scheme 6) [76].

## 3. Transformation of Lignin

**Figure 4:**
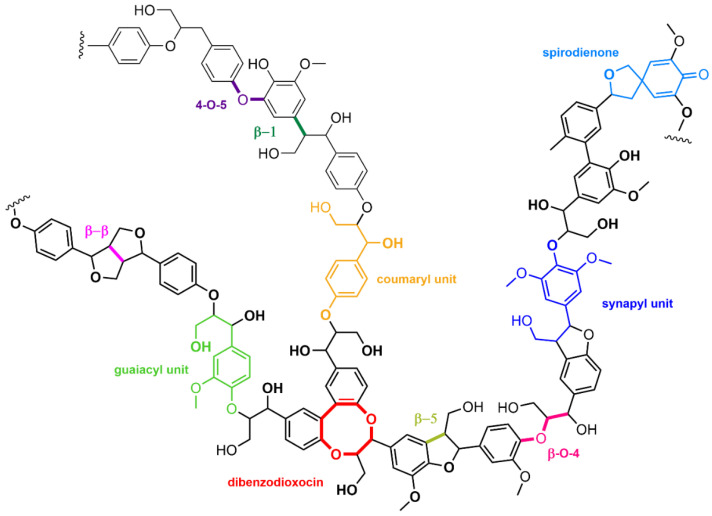
Example of lignin structure highlighting the diversity of constituting units and interunit bonds.

Lignin is another biopolymer constituting lignocellulose. Different from cellulose and hemicellulose, which are polysaccharides, lignin is constituted of aromatic units linked through a variety of bonds, each having a special reactivity. Indeed, the lignin structure (Figure 4) is obtained from the polymerization of three aromatic alcohols (coniferyl, sinapyl, and coumaryl), leading to a variety of compositions depending on the lignocellulose type (soft or hard wood, grass plants, vegetal wastes…).

Lignin can be a renewable source of aromatic chemicals, either for energy like toluene and for chemistry like vanillin, and their derivatives. Therefore, the number of studies has expanded rapidly during the past few years to valorise this biopolymer [77,78]. Its selective transformation represents a great challenge and in addition to the fact that lignin structure depends on its botanical origin, the extracted lignin is different than the native one because the extraction method can create new bonds between aromatic moieties. In consequence, and despite the fact that β-O-4 linkages correspond to half of the total interunit bonds, it appears initially difficult to consider general rules for catalytic lignin reactivity. Various methods of transformation into value-added molecules have been assessed in the literature, from enzymatic to chemo and organometallic catalysis. As we will see below, heterogeneous catalysis has also a role to play because it allows transformations using relatively demanding conditions leading to a specific reactivity of lignin.

### 3.1. Transformation of Lignin under Neutral Atmosphere

**Figure 5:**
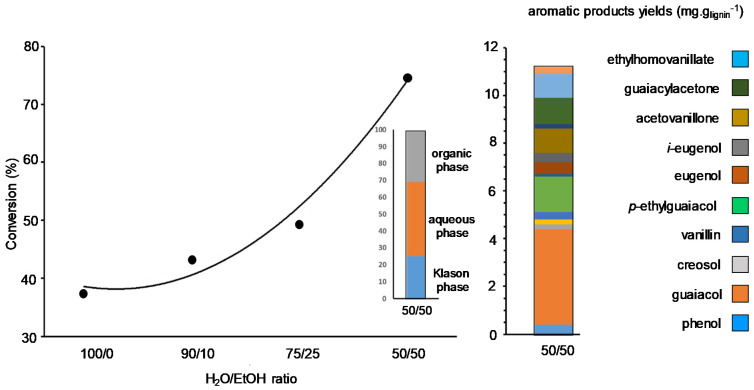
Influence of the water/ethanol mixture on lignin conversion with, after extraction, the phase repartition and the composition of the organic phase in the absence of catalyst.

Preliminary studies in the team consisted in comparing, under neutral atmosphere, the solvolysis of lignins from different origins and extraction methods: a spruce-lignin and a pine-lignin obtained from Kraft processes, a wheat straw-lignin obtained from Soda process, and a wheat-straw lignin obtained from Organosolv (CIMV^®^) process [79]. After full sample characterization by a variety of techniques, solvolysis was studied in EtOH-H_2_O under continuous flow conditions at 225 °C under 8 MPa of N_2_, preventing char and foam formation. Generally, after treating the reaction mixture three phases were obtained; the Klason phase corresponding to non-liquefied lignin, the aqueous phase containing mainly aliphatic water-soluble compounds, and the organic phase containing the target aromatic products. We showed the importance of the amount of EtOH (up to a 50 wt% mixture) on the content of monomeric organic products formed during the treatment. EtOH favours lignin conversion and stabilises organic products in which guaiacol was predominant due to β-O-4 bond cleavages (Figure 5). In these conditions, Kraft-spruce-lignin presented a higher reactivity (up to 76% of conversion) due to its natural solubility in EtOH-H_2_O while the other lignins necessitated the addition of K_2_CO_3_. Globally, it appears that product formation depends more on the origin of the lignins, rather than on their extraction process. When the reaction was performed on Kraft-spruce-lignin but in a closed reactor, the nature of the alcohol co-solvent had an impact on the amount of monomeric products. It increased from 0.8 to 4.8 wt% in the presence of iso-PrOH due to the formation of alkylated guaiacol species. After these non-catalyzed transformations, the influence of a supported metal catalyst under similar conditions using H_2_O-iso-PrOH solvents in an autoclave reactor was assessed. Pd/ZrO_2_ showed almost no influence on organic product formation, giving 4.4 wt% of aromatic products but increased significantly the total yield of organic products mainly oligomerics from 45 to 54 wt%. Note that when using Pd(OAC)_2_ as soluble catalyst, close results were obtained indicating in both cases the homogeneous nature of the reaction from soluble Pd species leached from Pd/ZrO_2_. In the last case we supposed that leached species performed primary depolymerization of lignin into monomers that further reacted on supported Pd species [80].

### 3.2. Transformation of Lignin under Oxidative Atmosphere

The reactivity of lignin in neutral conditions appears to give a very low amount of value-added molecules. Therefore, main catalytic approaches for the up-grading of lignin involve a reacting atmosphere, either reductive (H_2_) to produce deoxygenated aromatics for energy use, or oxidative (O_2_, air) to produce oxygenated aromatics for fine chemistry [81]. As far as heterogeneous catalysis is concerned, oxygenated aromatics can be obtained particularly with perovskites [82], mixed-metal oxides such as FeCoO as demonstrated by Barakat et al. [83], all giving global yields into aromatic aldehydes (vanillin, syringaldehyde…) in the 15–20 wt% range. Systems with supported metals have also been investigated with Co, Au, and Pd. Sales et al. have extensively studied Pd catalysts like Pd/γ-Al_2_O_3_, leading also around 10 wt% of aromatic aldehydes [84].

**Figure 6:**
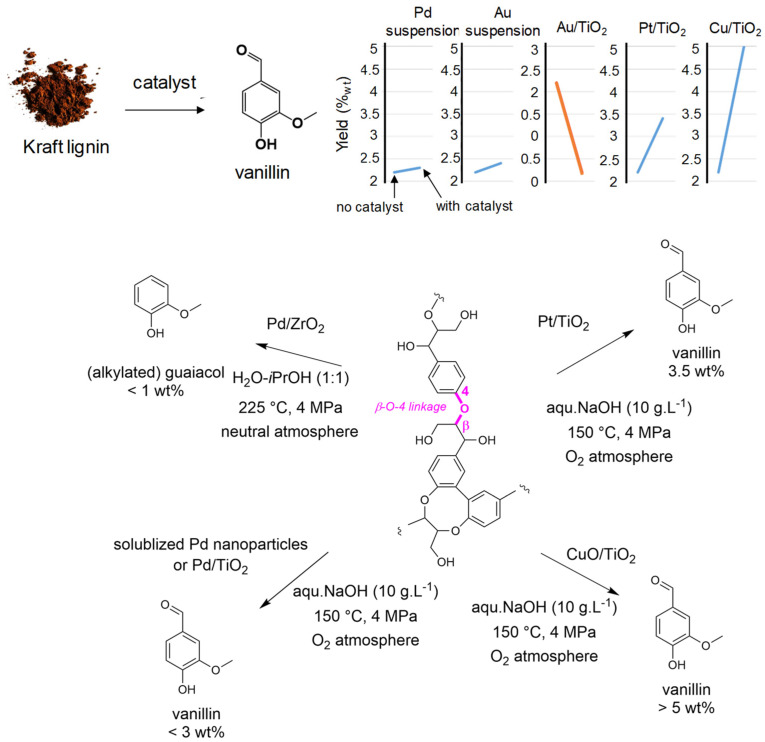
Catalyst development in C’Durable team for oxidative lignin depolymerization to aromatics, mainly vanillin.

Due to the molecular nature of the products obtained from the oxidative route, our team has been involved in the oxidative depolymerization of lignin for many years, particularly in water solvent. Pursuing with Pd systems, we prepared new Pd catalysts based on soluble PVP-stabilized or oxide supported nanoparticles (Pd/TiO_2_) (Figure 6). The homogeneous system was very efficient for the selective oxidation of benzyl alcohol as lignin models such as vanillic and veratryl alcohols into the corresponding aldehydes into mild conditions (80 °C, O_2_ flow) [85]. However, when applied to the oxidative depolymerization of a Kraft-pine-lignin [86], despite enhancing the lignin conversion compared to the absence of catalyst, the two Pd-systems did not improve the formation of the target products like vanillin and acetovanillone for which ones the yield remain low compared to catalyst free oxidative lignin depolymerization (i.e., for softwood Kraft lignins: from 2.4 wt% for non-catalyzed reaction to ca 2.2–2.5 wt% for Pd-catalyzed reactions) [87]. However, this reflects only an apparent lack of Pd-catalyst activity. Indeed, we demonstrated that these catalysts act more probably for oxidizing the Cα-OH (or Cγ-OH) junctions in β-O-4 bonds according to our studies on benzyl alcohol, before base catalyzed reactions take place (i.e., retro-aldol, tautomerization…) for breaking these bonds and delivering the expected aromatics [86]. Additionally, Pd-catalysts seem to favor recondensation reactions, thus enhancing the amount of Klason phase and, despite good reactivity in catalyzed reactions, did not deliver further aromatics.

Catalysts based on other metals, also known for their oxidation features have been employed (Figure 6). Here, we showed that Au/TiO_2_ had the tendency to further oxidize formed aromatics into volatile molecules. Thus very low yields into vanillin (0.2 wt%) were achieved. This result contrasts with that obtained when using Au nanoparticles in suspension that deliver results close to what was observed with Pd [86]. Supporting Au nanoparticles onto TiO_2_ seems to affect their activity. Pt/TiO_2_, unlike Pd catalysts, gave slightly enhanced yields of vanillin (from 2.4 wt% to 3.4 wt% for softwood Kraft lignin) and “syringaldehyde and vanillin” (from 4.6 wt% to 7.7 wt%, mainly on syringaldehyde). A kinetic study with this last catalyst indicated that it preserved the lignin substructures leading to a better yield of aromatics [88]. Later on, catalysts based on Cu have been prepared and used for the oxidative depolymerization of Kraft-pine-lignin. Compared to the non-catalyzed transformation, the presence of CuO/TiO_2_ did not affect the global lignin liquefaction (remaining around 70%) but increased the amount of valuable oxygenated aromatics especially vanillin formed up to 5 wt% in an autoclave reactor. In a continuous flow reactor this catalyst allows full lignin liquefaction with a higher aromatic molecule yield but without enhancing vanillin yield [89,90].

## 4. Transformation of Lignocellulose

As presented above, the selective transformation to molecules of interest of isolated lignocellulosic biopolymers is in itself difficult. Our team reported interesting and promising results during the past decade but progress to enhance our initial results was far from we could expect. There are possible reasons for that. Firstly, it is now well accepted that the lignocellulose fractionation processes into isolated cellulose and lignin modify significantly the structure of the native biopolymer and make it less reactive. This is clearly the case for all commercial samples used in the studies described above. For example, commercial celluloses are more crystalline than the native cellulose and are available with different characteristics (morphology, polymerization degree, crystallinity). These parameters influence the depolymerization and valorization efficiency. This explains why various pre-treatments are often applied to cellulose to enhance reactivity. The drawback is an increase of the heterogeneity of the solid reactant giving uncontrolled variabilities of the physico-chemical features. This also true for lignin, for which the origin and extraction method impact very much subsequent transformations. As a consequence, for both biopolymers data comparison has become very difficult for the scientific community. Secondly, a limitation relies on the difficulty to quantify the substrate conversion. Most of the studies on cellulose or lignin reactivity monitor the reaction progress based on the mass of solid residue. This can be mistaken in case of high molecular weight solid products (oligomers) formation and/or the use of a solid catalysts present in the residue (char, Klason lignin). Also, soluble oligomers present in product solution are usually not analyzed, hiding part of the products and distort data analysis. In consequence, it is of crucial importance to develop efficient and rapid analytical tools to accurately assess the biopolymers reactivity from a conversion and product yield point of view. In the case of cellulose, we attempted to improve this point using Infra-Red spectroscopy to quantify the unconverted solid to get real kinetics of conversion or applying liquid chromatography to analyse soluble oligosaccharide [67]. For instance, these reliable tools demonstrated the impact of solid catalysts WO_x_/ZrO_2_ on the initial rate of non-pre-treated cellulose conversion (see below).

The above comments give a clear idea on what can be the challenge with these biopolymers within a native lignocellulosic matrix. Indeed, if the main advantage is to have the biopolymers presumably more reactive, here come the potential issues of their interference during their respective catalytic transformation to a specific product, for instance impact on the accessibility to a heterogeneous catalyst, on poisoning of active sites by uncontrolled conversion of the other biopolymers. Nevertheless several groups are investigating the reactivity of raw biomass with heterogeneous catalysts. One can cite Fukuoka et al. [91] and Zhang et al. [92,93], who applied catalytic systems to wood reactivity to transform the carbohydrate biopolymers (cellulose and hemicellulose), into sugar-derived products giving close yields without apparent conversion of the lignin fraction. Wang et al. reported the valorization in a one-pot procedure with a single catalyst of all components of a lignocellulosic substrate into polyols from carbohydrate fractions, and cyclic alkanes from lignin [94]. While most of such studies try to valorize the carbohydrate fraction, releasing degraded lignin for challenging subsequent reactivity, Sels et al. developed the lignin-first concept of biomass transformation. This concept aims, during lignocellulose deconstruction, at lignin stabilization when depolymerization for example through hydrogenolysis for a more efficient catalytic valorization [95].

Another issue when the objective is the selective formation of a single product (or more modestly on a family of products), is to be able to convert one biopolymer keeping the others unchanged the most as possible, for parallel valorization. In this objective, again, analytical methods are very important when using lignocellulosic substrates of different nature depending on their origin and obtaining. Pertinent procedures for the preparation of the sample (drying, milling, sieving), the determination of its exact composition (cellulose, hemicellulose, lignin) before reaction and in solid reaction products are necessary in order to monitor the transformation progress. Among others, techniques for sample preparation, compositional analysis (acid hydrolysis combined to advanced Infra-Red spectroscopic methods), structural analysis (DRX, TGA, NMR) are particularly mastered in our research team [67,96]. The results we obtained on cellulose and lignin have participated to pave the way to study the reactivity of lignocellulose as a whole.

**Figure 7:**
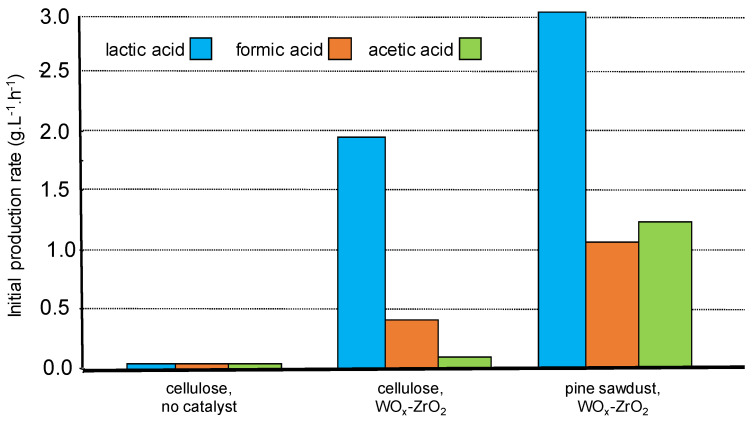
Comparison of cellulose and lignocellulose transformation in the presence of WO_x_-ZrO_2_ (water 65 g, wood 0.68 g, catalyst 0.3 g, 190 °C). Adapted from [63].

We have presented above the peculiar case of lactic acid formation from pine wood sawdust hydrothermal treatment in the presence of WO_x_-ZrO_2_ Lewis acid catalyst. Compared to commercial cellulose, results showed similar kinetic profiles indicated that the presence of other wood components like lignin did not affect the accessibility of the catalyst to the carbohydrate fractions (intrinsic hindrance), nor poison the active sites responsible for lactic acid formation. Moreover, a higher initial rate of product formation was observed from wood (Figure 7) certainly due to the different feature of cellulose (fiber morphology, lower crystallinity) and/or the contribution of hemicellulose in wood making its carbohydrate fraction more reactive [63]. Interestingly, it was shown by Infra-Red spectroscopy of the solid residue that the lignin component remained unchanged, certainly contributing to this result.

**Figure 8:**
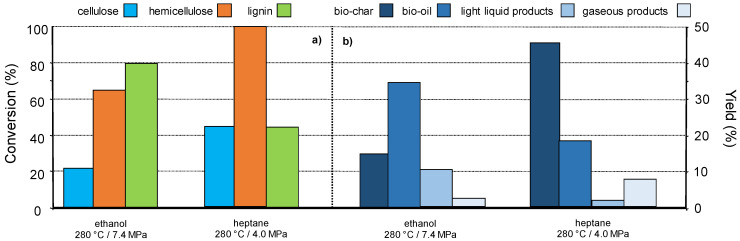
Pine wood (**a**) conversion and (**b**) products yields, in supercritical ethanol or supercritical heptane (solvent 27 g, wood 2.5 g, no catalyst, autogenous pressure, 1 h), in the absence of catalyst.

While the above result suggests that a fractionating pre-treatment is not always necessary to get specific molecules from lignocellulose [97,98], our team is investigating an original concept consisting in lignocellulose fragmentation in supercritical organic solvents in the presence of a catalyst. The supercritical conditions of an organic solvent may allow a partial selective conversion/liquefaction of lignocellulose components. Indeed, it is known that supercritical fluids possess solvation properties different than those under usual state. The solvation abilities of supercritical solvents can be tuned near the critical point by small change in the temperature, the fluid density. The composition and nature of the supercritical fluid (pure solvent or mixture) is also of prime importance regarding the chemical nature of the solid substrate to be solubilized. Thus, this methodology relies on the assumption to selectively solubilize one of the wood components, (hemi)cellulose vs. lignin by choosing the nature of the supercritical fluid and conditions. As an example, we investigated the behaviour of pine wood sawdust in ethanol and heptane in supercritical state (critical points 241 °C and 6.1 MPa, 267 °C and 27 MPa, respectively) at 280 °C in autoclave reactor. Figure 8a shows that depending on the supercritical fluid, the biopolymers conversions are different. For example, lignin was more converted than hemicellulose in ethanol at 280 °C (10 MPa), and this was the contrary in heptane. In heptane, under the same conditions, hemicellulose was completely converted vs. 40 wt% for cellulose and lignin. The product families were also different, giving mainly bio-oils in ethanol (35 wt%) and bio-char in heptane (40 wt%) (Figure 8b) [99].

**Figure 9:**
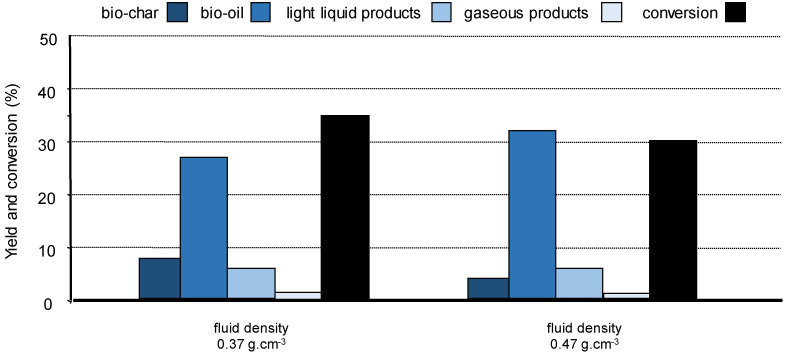
Pine wood conversion in supercritical ethanol at 250 °C. Influence of the fluid density on the yields of bio-char, bio-oils, light products, and gases, in the absence of catalyst. Adapted from [100].

For a specific solvent, fine condition tuning such as the fluid density influences wood conversion and product distribution. Treating pine wood with supercritical ethanol of higher density led to more bio-oil and less bio-char, most likely explained by clustering effect which may favour reaction between ethanol and the biopolymer fragments at the expense of their recondensation (Figure 9) [100]. These results confirm that (partial) selective fractionation of lignocellulose is feasible by using a solvent of adequate polarity and functionality and playing around the reaction conditions, especially near the critical point of the solvent.

In the absence of catalyst, the selective formation of liquid light products was clearly not favoured, and the conversion/liquefaction mainly produced bio-oil up to 30 wt% of initial substrate, using supercritical ethanol for example.

**Figure 10:**
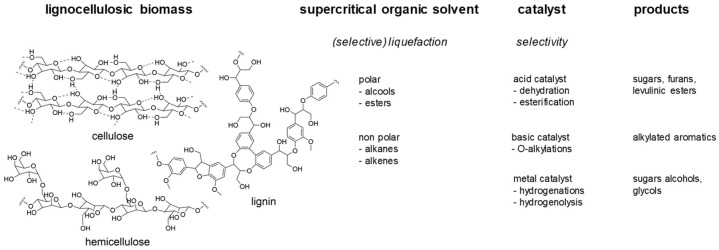
Strategy for lignocellulose fractionation in supercritical solvents in the presence of a catalyst.

This is why the transformation strategy can also include the presence of a catalyst, at the early stage of the fractionation, in order to drive a liquefaction into the formation of a specific product. Some possibilities are presented in Figure 10. Studies are currently in progress in the team to determine the influence of the characteristics of various solvents and catalyst on the sequential lignocellulose component liquefaction and the nature and composition of the products [101].

**Figure 11:**
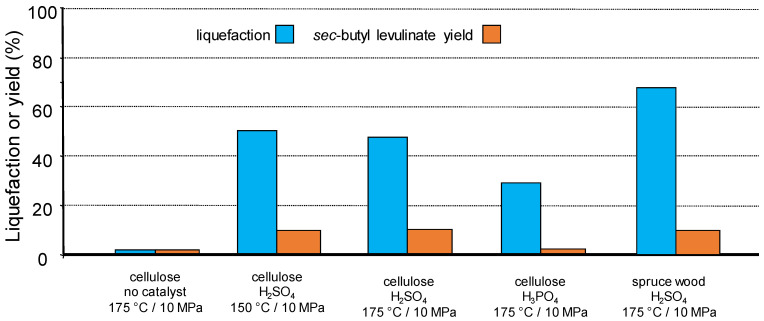
Biomass liquefaction and sec-butyl levulinate formation after treatment under super-critical 2-butene (flow 0.1 mL·min^−1^, reactant 2 g, 10 MPa He, 1.5 h).

An additional possibility to perform more selective transformations is the use of a semi-continuous flow reactor in which the solvent circulates through a fixed-bed formed of reactant and catalyst. Advantages are the possibility to finely and independently tune the temperature and pressure and to control more precisely the residence time under certain conditions. This may impact favourably the selectivity of the transformation, for example, by avoiding modification of products as observed in autoclave reactors [99], or limiting successive reactions. We initiated this concept by reacting a fixed-bed of cellulose or spruce wood maintained in an open tubular reactor (beforehand wet-impregnated with 0.1N solution of mineral acid) through the percolation of supercritical 2-butene (critical points 146 °C and 4.0 MPa) under various conditions. A yield up to 13 mol% of sec-butyl levulinate (based on cellulose content) was achieved through a protolysis and esterification sequence (Figure 11) [102,103]. Note the importance of the presence and nature of adsorbed mineral acid on the efficiency of the reaction under these conditions.

## 5. Conclusions

This review describes the main advances obtained in the C’Durable team at IRCELYON in the area of molecular transformation of lignocellulose biopolymers for the last decade. Our goal is to form value-added molecules, using sustainable processes as much as possible. In this view the use of heterogeneous catalysis is a tool with definitive advantages to drive the reactivity towards robust and selective formation of products of interest. To date we have been mainly working on cellulose and lignin transformation applying an approach consisting in studying the non-catalyzed transformation, then the impact of heterogeneous catalysts on the conversion and product formation. We had the chance to face with many problematics that can be encountered in this field of research, giving us a deep scientific and technical know-how to develop catalytic processes for the valorization of this bioresource. Henceforth we try at present to apply all this knowledge to efficient transformation of raw lignocellulose, which we believe is going to be one of the future challenges in the field of green chemistry for a sustainable development.

## Data Availability

All data can be recovered from the cited literature.

## References

[1] Luterbacher J.S., Martin Alonso D., Dumesic J.A. (2014). Targeted chemical upgrading of lignocellulosic biomass to platform molecules. Green Chem..

[2] Gallezot P. (2012). Conversion of biomass to selected chemical products. Chem. Soc. Rev..

[3] Sudarsanam P., Zhong R., Van den Bosch S., Coman S.M., Parvulescu V.I., Sels B.F. (2018). Functionalised heterogeneous catalysts for sustainable biomass valorisation. Chem. Soc. Rev..

[4] De S., Dutta S., Saha B. (2016). Critical design of heterogeneous catalysts for biomass valorization: Current thrust and emerging prospects. Catal. Sci. Technol..

[5] Besson M., Gallezot P., Pinel C. (2014). Conversion of biomass into chemicals over metal catalysts. Chem. Rev..

[6] Delidovich I., Leonhard K., Palkovits R. (2014). Cellulose and hemicellulose valorisation: An integrated challenge of catalysis and reaction engineering. Energy Environ. Sci..

[7] Fogassy G., Ke P., Figueras F., Cassagnau P., Rouzeau S., Courault V., Gelbard G., Pinel C. (2011). Catalyzed ring opening of epoxides: Application to bioplasticizers synthesis. Appl. Catal. A.

[8] Fabiano D.P., Hamad B., Cardoso D., Essayem N. (2010). On the understanding of the remarkable activity of template-containing mesoporous molecular sieves in the transesterification of rapeseed oil with ethanol. J. Catal..

[9] Hamad B., Lopes de Souza R.O., Sapaly G., Carneiro Rocha M.G., Pries de Oliveira P.G., Gonzalez W.A., Andrade Sales E., Essayem N. (2008). Transesterification of rapeseed oil with ethanol over heterogeneous heteropolyacids. Catal. Commun..

[10] Morin P., Hamad B., Sapaly G., Carneiro Rocha M.G., Pries de Oliveira P.G., Gonzalez W.A., Andrade Sales E., Essayem N. (2007). Transesterification of rapeseed oil with ethanol. I. Catalysis with homogeneous Keggin heteropolyacids. Appl. Catal. A.

[11] Mesnager J., Quettier C., Lambin A., Rataboul F., Perrard A., Pinel C. (2010). Telomerization of butadiene with starch in water: Role of the surfactants. Green Chem..

[12] Mesnager J., Quettier C., Lambin A., Rataboul F., Pinel C. (2009). Telomerization of butadiene with starch under mild conditions. ChemSusChem.

[13] Sorokin A.B., Kachkarova-Sorokina S.L., Donze C., Pinel C., Gallezot P. (2004). From native starch to hydrophilic and hydrophobic products: A catalytic approach. Top. Catal..

[14] Donze C., Pinel C., Gallezot P., Taylor P.L. (2002). Palladium-catalyzed telomerization of butadiene with starch. Adv. Synth. Catal..

[15] Braga T.P., Essayem N., Valentini A. (2015). Synthesis of Cu-M_x_O_y_/Al_2_O_3_ (M = Fe, Zn, W or Sb) catalysts for the conversion of glycerol to acetol: Effect of texture and acidity of the supports. RSC Adv..

[16] Ftouni J., Villandier N., Auneau F., Besson M., Djakovitch L., Pinel C. (2015). From glycerol to lactic acid under inert conditions in the presence of platinum-based catalysts: The influence of support. Catal. Today.

[17] Garcia R., Besson M., Gallezot P. (1995). Chemoselective catalytic oxidation of glycerol with air on platinum metals. Appl. Catal. A.

[18] Auneau F., Noel S., Aubert G., Besson M., Djakovitch L., Pinel C. (2011). On the role of the atmosphere in the catalytic glycerol transformation over iridium-based catalysts. Catal. Commun..

[19] Auneau F., Michel C., Delbecq F., Pinel C., Sautet P. (2011). Unravelling the mechanism of glycerol hydrogenolysis over rhodium catalyst through combined experimental-theoretical investigations. Chem. Eur. J..

[20] Essayem N., Lopes de Souza R.O., Hamad B., Sapaly G., Pries de Oliveira P.G., de Aurojo Gonzales W. (2009). Simultaneous Glyceride Transesterification-Glycerol Etherification for Manufacture of Biodiesel and Fuel Additives.

[21] Chaminand J., Djakovitch L., Gallezot P., Marion P., Pinel C., Rosier C. (2004). Glycerol hydrogenolysis on heterogeneous catalysts. Green Chem..

[22] Abdouli I., Eternot M., Dappozze F., Guillard C., Essayem N. (2021). Comparison of hydrothermal and photocatalytic conversion of glucose with commercial TiO_2_: Superficial properties-activities relationships. Catal. Today.

[23] Riviere M., Perret N., Delcroix D., Cabiac A., Pinel C., Besson M. (2018). Solvent effect in hydrogenolysis of xylitol over bifunctional Ru/MnO/C catalysts under alkaline-free conditions. ACS Sustain. Chem. Eng..

[24] Doiseau A.C., Rataboul F., Burel L., Essayem N. (2014). Synergy effect between solid acid catalysts and concentrated carboxylic acids solutions for efficient furfural production from xylose. Catal. Today.

[25] Auneau F., Berchu M., Aubert G., Pinel C., Besson M., Todaro D., Bernardi M., Ponsetti T., Di Felice R. (2014). Exploring the reaction conditions for Ru/C catalyzed selective hydrogenolysis of xylitol alkaline aqueous solutions to glycols in a trickle-bed reactor. Catal. Today.

[26] Karaki M., Karout A., Toufaily J., Rataboul F., Essayem N., Lebeau B. (2013). Synthesis and characterization of acidic ordered mesoporous organosilica SBA-15: Application to the hydrolysis of cellobiose and insight into the stability of the acidic functions. J. Catal..

[27] Souza R.O.L., Fabiano D.P., Feche C., Rataboul F., Cardoso D., Essayem N. (2012). Glucose-fructose isomerisation promoted by basic hybrid catalysts. Catal. Today.

[28] Perrard A., Gallezot P., Joly J.-P., Durand R., Baljou C., Coq B., Trens P. (2007). Highly efficient metal catalysts supported on activated carbon cloths: A catalytic application for the hydrogenation of D-glucose to D-sorbitol. Appl. Catal. A.

[29] Gallezot P., Nicolaus N., Fleche G., Fuertes P., Perrard A. (1998). Glucose hydrogenation on ruthenium catalysts in a trickle-bed reactor. J. Catal..

[30] Dechamp N., Gamez A., Perrard A., Gallezot P. (1995). Kinetics of glucose hydrogenation in a trickle-bed reactor. Catal. Today.

[31] Besson M., Lahmer F., Gallezot P., Fuertes P., Fleche G. (1995). Catalytic oxidation of glucose on bismuth-promoted palladium catalysts. J. Catal..

[32] Ait Rass H., Essayem N., Besson M. (2015). Selective aerobic oxidation of 5-HMF into 2,5-furandicarboxylic acid with Pt catalysts supported on TiO_2_- and ZrO_2_-based supports. ChemSusChem.

[33] Ait Rass H., Essayem N., Besson M. (2013). Selective aqueous phase oxidation of 5-hydroxymethylfurfural to 2,5-furandicarboxylic acid over Pt/C catalysts: Influence of the base and effect of bismuth promotion. Green Chem..

[34] Lopes de Souza R.O., Rataboul F., Essayem N. (2012). Hydroxymethylfurfural (5-HMF) production from hexoses: Limits of heterogeneous catalysis in hydrothermal conditions and potential of concentrated aqueous organic acids solutions as reactive solvent system. Challenges.

[35] Olivier-Bourbigou H., Chizallet C., Dumeignil F., Fongarland P., Geantet C., Granger P., Launay F., Löfberg A., Massiani P., Maugé F. (2017). The pivotal role of catalysis in France: Selected examples of recent advances and future prospects. ChemCatChem.

[36] Klemm D., Heublein B., Fink H.-P., Bohn A. (2005). Cellulose: Fascinating biopolymer and sustainable raw material. Angew. Chem. Int. Ed..

[37] Cabiac A., Guillon E., Chambon F., Pinel C., Rataboul F., Essayem N. (2011). Cellulose reactivity and glycosidic bond cleavage in aqueous phase by catalytic and non catalytic transformations. Appl. Catal. A.

[38] Lanzafame P., Temi D.M., Perathoner S., Spadaro A.N., Centi G. (2012). Direct conversion of cellulose to glucose and valuable intermediates in mild reaction conditions over solid acid catalysts. Catal. Today.

[39] Pang J., Wang A., Zheng M., Zhang T. (2010). Hydrolysis of cellulose into glucose over carbons sulfonated at elevated temperatures. Chem. Commun..

[40] Suganuma S., Nakajima K., Kiyano M., Yamaguchi D., Kato H., Hayashi S., Hara M. (2008). Hydrolysis of cellulose by amorphous carbon bearing SO_3_H, COOH, and OH groups. J. Am. Chem. Soc..

[41] Onda A., Ochi T., Yanagisawa K. (2008). Selective hydrolysis of cellulose into glucose over solid acid catalysts. Green Chem..

[42] Van de Vyver S., Peng L., Geboers J., Schepers H., de Clippel F., Gommes C.J., Goderis B., Jacobs P.A., Sels B.F. (2010). Sulfonated silica/carbon nanocomposites as novel catalysts for hydrolysis of cellulose to glucose. Green Chem..

[43] Vilcocq L., Castilho P.C., Carvalheiro F., Duarte L.C. (2014). Hydrolysis of oligosaccharides over solid acid catalysts: A review. ChemSusChem.

[44] Rinaldi R., Schüth F. (2009). Acid hydrolysis of cellulose as the entry point into biorefinery schemes. ChemSusChem.

[45] Antonetti C., Licursi D., Fulignati S., Valentini G., Raspolli Galletti A.M. (2016). New frontiers in the catalytic synthesis of levulinic acid: From sugars to raw and waste biomass as starting feedstock. Catalysts.

[46] Rinaldi R., Meine N., vom Stein J., Palkovits R., Schüth F. (2010). Which controls the depolymerization of cellulose in ionic liquids: The solid acid catalyst or cellulose?. ChemSusChem.

[47] De Oliveira Vigier K., Chatel G., Jérôme F. (2015). Contribution of deep eutectic solvents for biomass processing: Opportunities, challenges, and limitations. ChemCatChem.

[48] Jérôme F., Chatel G., De Oliveira Vigier K. (2016). Depolymerization of cellulose to processable glucans by non-thermal technologies. Green Chem..

[49] Fukuoka A., Dhepe P.L. (2006). Catalytic conversion of cellulose into sugar alcohols. Angew. Chem. Int. Ed..

[50] Ruppert A.M., Weinberg K., Palkovits R. (2012). Hydrogenolysis goes bio: From carbohydrates and sugar alcohols to platform chemicals. Angew. Chem. Int. Ed..

[51] Kobayashi H., Komanoya T., Guha S.K., Hara K., Fukuoka A. (2011). Conversion of cellulose into renewable chemicals by supported metal catalysis. Appl. Catal. A.

[52] Ji N., Zhang T., Zheng M., Wang A., Wang H., Wang X., Chen J.G. (2008). Direct catalytic conversion of cellulose into ethylene glycol using nickel-promoted tungsten carbide catalysts. Angew. Chem. Int. Ed..

[53] Jollet V., Chambon F., Rataboul F., Cabiac A., Pinel C., Guillon E., Essayem N. (2009). Non-catalyzed and Pt/γ-Al_2_O_3_-catalyzed hydrothermal cellulose dissolution-conversion: Influence of the reaction parameters and analysis of the unreacted cellulose. Green Chem..

[54] Möller  M., Nilges  P., Harnisch F., Schröder U. (2011). Subcritical water as reaction environment: Fundamentals of hydrothermal biomass transformation. ChemSusChem.

[55] Chen P., Shrotri A., Fukuoka A. (2021). Synthesis of cello-oligosaccharides by depolymerization of cellulose: A review. Appl. Catal. A.

[56] Fongarland P., Essayem N., Rataboul F. (2017). Noncatalyzed liquefaction of celluloses in hydrothermal conditions: Influence of reactant physicochemical characteristics and modeling studies. Ind. Eng. Chem. Res..

[57] Chambon F., Essayem N., Rataboul F., Pinel C., Cabiac A., Guillon E. (2012). Process for Converting Cellulose or Lignocellulosic Biomass Using Stable Non-Zeolite Solid Lewis Acids Based on Tin or Antimony Alone or as a Mixture.

[58] Chambon F., Essayen N., Rataboul F., Pinel C., Cabiac A., Guillon E. (2011). Process for Conversion of Lignocellulosic Biomass or Cellulose by Using Supported Tungsten-Based Solid Lewis Acids as Catalysts.

[59] Nguyen V.C., Dandach A., Vu T.T.H., Fongarland P., Essayem N. (2019). ZrW catalyzed cellulose conversion in hydrothermal conditions: Influence of the calcination temperature and insights on the nature of the active phase. Molecular Catal..

[60] Chambon F., Rataboul F., Pinel C., Cabiac A., Guillon E., Essayem N. (2011). Cellulose hydrothermal conversion promoted by heterogeneous Brønsted and Lewis acids: Remarkable efficiency of solid Lewis acids to produce lactic acid. Appl. Catal. B.

[61] Mäki-Arvela P., Simakova I.L., Salmi T., Murzin D.Y. (2014). Production of lactic acid/lactates from biomass and their catalytic transformations to commodities. Chem. Rev..

[62] West R.M., Holm M.S., Saravanamurugan S., Xiong J., Beversdorf Z., Taarning E., Christensen C.H. (2010). Zeolite H-USY for the production of lactic acid and methyl lactate from C3-sugars. J. Catal..

[63] Swesi Y., Nguyen C., Vu T.T.H., Rataboul F., Eternot M., Fongarland P., Essayem N. (2017). Direct solid Lewis acid catalyzed wood liquefaction into lactic acid: Kinetic evidences that wood pretreatment might not be a prerequisite. ChemCatChem.

[64] Chambon F., Rataboul F., Pinel C., Cabiac A., Guillon E., Essayem N. (2013). Cellulose conversion with tungstated-alumina-based catalysts: Influence of the presence of platinum and mechanistic studies. ChemSusChem.

[65] Chambon F., Essayem N., Rataboul F., Pinel C., Cabiac A., Guillon E. (2012). Process for Conversion of Lignocellulosic Biomass or Cellulose Using Solid Lewis Acid Catalysts Containing an Oxide of Tungsten and a Metal from Groups 8 to 11.

[66] Chambon F., Rataboul F., Pinel C., Cabiac A., Guillon E., Essayem N. (2015). Conversion of cellulose to 2,5-hexanedione using tungstated zirconia in hydrogen atmosphere. Appl. Catal. A.

[67] Nguyen V.C., Bui N.Q., Eternot M., Vu T.T.H., Fongarland P., Essayem N. (2018). Kinetic of ZrW catalyzed cellulose hydrothermal conversion: Deeper understanding of reaction pathway via analytic tools improvement. Mol. Catal..

[68] Liu Y., Li G., Hu Y., Wang A., Lu F., Zou J.-J., Cong Y., Li N., Zhang T. (2019). Integrated conversion of cellulose to high-density aviation fuel. Joule.

[69] Démolis A., Essayem N., Rataboul F. (2014). Synthesis and applications of alkyl levulinates. ACS Sustain. Chem. Eng..

[70] Garves K. (1988). Acid catalyzed degradation of cellulose in alcohols. J. Wood Chem. Technol..

[71] Rataboul F., Essayem N. (2011). Cellulose reactivity in supercritical methanol in the presence of solid acid catalysts: Direct synthesis of methyl-levulinate. Ind. Eng. Chem. Res..

[72] Démolis A., Eternot M., Essayem N., Rataboul F. (2016). Influence of butanol isomers on the reactivity of cellulose towards the synthesis of butyl levulinates catalyzed by liquid and solid acid catalysts. New J. Chem..

[73] Guerbuez E.I., Alonso D.M., Bond J.Q., Dumesic J.A. (2011). Reactive extraction of levulinate esters and conversion to γ-valerolactone for production of liquid fuels. ChemSusChem.

[74] Démolis A., Eternot M., Essayem N., Rataboul F. (2017). New insights into the reactivity of biomass with butenes for the synthesis of butyl levulinates. ChemSusChem.

[75] Strappaveccia G., Luciani L., Bartollini E., Marrocchi A., Pizzo F., Vaccaro L. (2015). γ-Valerolactone as an alternative biomass-derived medium for the Sonogashira reaction. Green Chem..

[76] Marcel R., Durillon T., Djakovitch L., Fache F., Rataboul F. (2019). First example of the use of biosourced alkyl levulinates as solvents for synthetic chemistry: Application to the heterogeneously catalyzed Heck coupling. ChemistrySelect.

[77] Sun Z., Fridrich B., de Santi A., Elangovan S., Barta K. (2018). Bright side of lignin depolymerization: Toward new platform chemicals. Chem. Rev..

[78] Zakzeski J., Bruijnincx P.C.A., Jongerius A.L., Weckhuysen B.M. (2010). The catalytic valorization of lignin for the production of renewable chemicals. Chem. Rev..

[79] Sebhat W., El-Roz A., Crepet A., Ladaviere C., Perez D.D.S., Mangematin S., Cabral Almada C., Vilcocq L., Djakovitch L., Fongarland P. (2020). Comparative study of solvolysis of technical lignins in flow reactor. Biomass Conv. Biorefin..

[80] Sebhat W., El Roz A., Fongarland P., Vilcocq L., Djakovitch L. (2021). Catalytic liquefaction of Kraft lignin with solvothermal approach. Catalysts.

[81] Bourbiaux D., Pu J., Rataboul F., Djakovitch L., Geantet C., Laurenti D. (2021). Reductive or oxidative catalytic lignin depolymerization: An overview of recent advances. Catal. Today.

[82] Zhang J., Deng H., Lin L. (2009). Wet aerobic oxidation of lignin into aromatic aldehydes catalyzed by a perovskite-type-oxide: LaFe_1-x_Cu_x_O_3_ (x = 0, 0.1, 0.2). Molecules.

[83] Hdidou L., Kouisni B., Manoun H., Hannache H., Solhy A., Barakat A. (2018). Oxidative conversion of lignin over cobalt-iron mixed oxides prepared via the alginate gelation. Catal. Commun..

[84] Sales F.G., Maranhao L.C.A., Lima filho N.M., Abreu C.A.M. (2006). Kinetic evaluation and modeling of lignin catalytic wet oxidation to selective production of aromatic aldehydes. Ind. Eng. Chem. Res..

[85] Bourbiaux D., Mangematin S., Djakovitch L., Rataboul F. (2021). Selective aerobic oxidation of benzyl alcohols with palladium(0) nanoparticles suspension in water. Catal. Lett..

[86] Bourbiaux D., Xu Y., Goc F., Fongarland P., Philippe R., Aubert G., Aymonier C., Rataboul F., Djakovitch L. (2021). Investigating (pseudo)-heterogeneous Pd-catalysts for Kraft lignin depolymerisation under mild aqueous basic conditions. Catalysts.

[87] Cabral Almada C., Kazachenko A., Fongarland P., Da Silva Perez D., Kuznetsov B.N., Djakovitch L. (2020). Oxidative depolymerization of lignins for producing aromatics: Variation of botanical origin and extraction methods. Biomass Conv. Biorefin..

[88] Cabral Almada C., Kazachenko A., Fongarland P., Da Silva Perez D., Kuznetsov B.N., Djakovitch L. (2021). Supported-metal catalysts in upgrading lignin to aromatics by oxidative depolymerization. Catalysts.

[89] Hernandez Manas A., Mangematin S., Vilcocq L., Fongarland P., Djakovitch L. From technical lignins to aromatics: A study on oxidative depolymerization in batch and continuous reactor. Proceedings of the 5th International Congress on Catalysis for Biorefineries (CatBior-5).

[90] Hernandez Manas A., Zhou S., Djakovitch L., Mangematin S., Liu S., Vilcocq L., Fongarland P. Lignin depolymerization in oxidative conditions: From batch to continuous reactor. Proceedings of the 4th Iberoamerican Congress on Biorefineries.

[91] Yamaguchi A., Sato O., Mimura N., Hirosaki Y., Kobayashi H., Fukuoka A., Shirai M. (2014). Direct production of sugar alcohols from wood chips using supported platinum catalysts in water. Catal. Commun..

[92] Liu Q., Zhang T., Liao Y., Cai C., Tan J., Wang T., Qiu S., He M., Ma L. (2017). Production of C5/C6 sugar alcohols by hydrolytic hydrogenation of raw lignocellulosic biomass over Zr based solid acids combined with Ru/C. ACS Sustain. Chem. Eng..

[93] Li C., Zheng M., Wang A., Zhang T. (2012). One-pot catalytic hydrocracking of raw woody biomass into chemicals over supported carbide catalysts: Simultaneous conversion of cellulose, hemicellulose and lignin. Energy Environ. Sci..

[94] Li X., Guo T., Xia Q., Liu X., Wang Y. (2018). One-pot catalytic transformation of lignocellulosic biomass into alkylcyclohexanes and polyols. ACS Sustain. Chem. Eng..

[95] Renders T., Van den Bosch S., Koelewijn S.-K., Sels B.F. (2017). Lignin-first biomass fractionation: The advent of active stabilisation strategies. Energy Environ. Sci..

[96] Bui N.Q., Fongarland P., Rataboul F., Dartiguelongue C., Charon N., Vallée C., Essayem N. (2015). FTIR as a simple tool to quantify unconverted lignin from chars in biomass liquefaction process: Application to SC ethanol liquefaction of pine wood. Fuel Process. Technol..

[97] Putro J.N., Soetaredjo F.E., Lin S.-Y., Ju Y.-H., Ismadji S. (2016). Pretreatment and conversion of lignocellulose biomass into valuable chemicals. RSC Adv..

[98] Silveira M.H.L., Morais A.R.C., da Costa Lopes A.M., Olekszyszen D.N., Bogel-Łukasik R., Andreaus J., Pereira Ramos L. (2015). Current pretreatment technologies for the development of cellulosic ethanol and biorefineries. ChemSusChem.

[99] Eternot M., Rataboul F., Essayem N. SC organic solvents coupled to heterogeneous catalysis: A unique tool for selective wood components liquefaction into chemicals. Proceedings of the 4th International Congress on Catalysis for Biorefineries (CatBior-4).

[100] Bui N.Q., Fongarland P., Rataboul F., Dartiguelongue C., Charon N., Vallée C., Essayem N. (2018). Controlled pinewood fractionation with supercritical ethanol: A prerequisite toward pinewood conversion into chemicals and biofuels. Comptes Rendus Chim..

[101] Eternot M., Rataboul F., Essayem N. Sequential wood components liquefaction using supercritical organic solvents mixtures implemented in a flowthrough reactor. Proceedings of the 18th European Meeting on Supercritical Fluids (Webconference).

[102] Essayem N., Sapaly G., Eternot M., Rataboul F. (2014). Method for Preparing Levulinic Acid Esters.

[103] Rataboul F., Sapaly G., Eternot M., Essayem N. Acid-catalyzed direct formation of sec-butyl-levulinate from the reaction of cellulose with 2-butene. Proceedings of the 15th International Congress on Catalysis (ICC-15).

